# Homozygous Wildtype of XPD K751Q Polymorphism Is Associated with Increased Risk of Nasopharyngeal Carcinoma in Malaysian Population

**DOI:** 10.1371/journal.pone.0130530

**Published:** 2015-06-18

**Authors:** Munn-Sann Lye, Shaneeta Visuvanathan, Pei-Pei Chong, Yoke-Yeow Yap, Chin-Chye Lim, Eng-Zhuan Ban

**Affiliations:** 1 Department of Community Health, Faculty of Medicine and Health Sciences, Universiti Putra Malaysia, Serdang, Selangor, Malaysia; 2 Department of Biomedical Sciences, Faculty of Medicine and Health Sciences, Universiti Putra Malaysia, Serdang, Selangor, Malaysia; 3 Department of Surgery, Faculty of Medicine and Health Sciences, Universiti Putra Malaysia, Serdang, Selangor, Malaysia; 4 National Cancer Institute, Ministry of Health Malaysia, Putrajaya, Malaysia; National Cancer Center, JAPAN

## Abstract

The xeroderma pigmentosum group D (XPD) gene encodes a DNA helicase, an important component in transcription factor IIH (TFIIH) complex. XPD helicase plays a pivotal role in unwinding DNA at the damaged region during nucleotide excision repair (NER) mechanism. Dysfunctional XPD helicase protein from polymorphic diversity may contribute to increased risk of developing cancers. This study aims to determine the association between XPD K751Q polymorphism (rs13181) and risk of nasopharyngeal carcinoma (NPC) in the Malaysian population. In this hospital-based matched case-control study, 356 controls were matched by age, gender and ethnicity to 356 cases. RFLP-PCR was used to genotype the XPD K751Q polymorphism. A significant association was observed between XPD K751Q polymorphism and the risk of NPC using conditional logistic regression. Subjects with homozygous Lys/Lys (wildtype) genotype have 1.58 times higher odds of developing NPC compared to subjects with recessive combination of heterozygous Lys/Gln and homozygous Gln/Gln genotypes (OR = 1.58, 95% CI = 1.05–2.38 p = 0.028) adjusted for cigarette smoking, alcohol and salted fish consumption. Our data suggests that Lys/Lys (wildtype) of XPD K751Q contributes to increased risk of NPC in the Malaysian population.

## Introduction

Nasopharyngeal carcinoma (NPC) originates from the epithelial lining of the nasopharynx. In most parts of the world, NPC is an uncommon cancer. The incidence proportion of NPC in the United States is as low as 1 per 100,000 population [1] whereas in Southeast Asia (mainly in Malaysia, Singapore and Indonesia) it averages 6.5 per 100,000 population [2]. According to the National Cancer Statistics Malaysia 2007, incidence proportion of NPC in Malaysia was 6.4 per 100,000 population in males and 2.3 per 100,000 population in females [3]. Several postulations have been made to explain this disparity in NPC incidence in different parts of the world. Etiological factors such as genetic susceptibility, consumption of high-salt-content-preserved food and cigarette smoking were associated with increased risk of NPC [4–6]. Exposures to nitrosamines from salted fish could lead to the formation of harmful DNA adducts [7]. Accumulation of DNA adducts in normal healthy cells without proper DNA repair could result in genomic instability [8]. DNA repair systems play an important role in preserving the human genome from genotoxic stress exerted by both exogenous as well as endogenous carcinogens such as reactive oxygen species and DNA single strand breaks [9]. Single nucleotide polymorphisms (SNPs) in DNA repair genes have been extensively studied in relation to cancer development due to their potential effect on the maintenance of genomic integrity [10] and some of these have been found to be associated with increased risk of developing cancers. However, the role of these SNPs remains largely unknown in relation to NPC carcinogenesis.

Human XPD gene is an important component in transcription factor IIH (TFIIH) complex and is responsible for encoding an ATP-dependent 5’-3’ DNA helicase protein, which is 761 amino acids long in sequence and has a molecular weight of 86,909 Da [11]. XPD helicase consists of 4 domains namely helicase domains 1 and 2 (where carboxy terminal domain, CTD is located), Arch and FeS cluster-containing domains [12]. TFIIH is the main protein complex involved in eukaryotic NER mechanism [13] and is made up of XPD helicase and 2 sub-complexes involving 9 subunits, namely XPB, p62, p52, p44, p34 and p8 combined that form the core, and also cdk7, cdk-activating kinase assembly factor 1 (MAT1) and cyclin H bound together forming another sub-complex known as cdk-activating kinase (CAK). XPD helicase is an indispensable member of TFIIH complex because the helicase bridges both sub-complexes of TFIIH together [14]. XPD links p44 and MAT1 [15–17] via the binding of MAT1 to the XPD helicase’s Arch domain while p44 interacts with CTD of the same helicase. With XPD bridging both of the sub-complexes, functional TFIIH plays a pivotal role in DNA repair [18–19]. The role of NER mechanism in the removal of helix-distorting bulky DNA adducts is important in maintaining a low level of DNA damage in human cells. Smaller DNA adducts such as cisplatin and ultra-violet exposure-related photoproducts are also removed by NER [20]. The binding of XPD to CAK complex negatively regulates its helicase activity whereas interaction with p44 enhances the aforementioned activity of XPD [21–22]. XPD gene polymorphisms have been observed in almost every region of the helicase structure [23]. R156R (rs238406), D312N (rs1799793) as well as K751Q (rs13181) are examples of notable cancer-associated polymorphisms and they are located in HD1, Arch and CTD domains of XPD helicase respectively [24]. These single nucleotide polymorphisms (SNPs) have been studied extensively and been implicated in candidate gene studies. While several studies reported significant associations of these SNPs in cancer development [25–31], others did not [32–34]. We focused on K751Q polymorphism because this SNP is located in the XPD-p44 interacting C terminus region, and interaction with p44 is important for XPD helicase activity.

## Materials and Methods

### Ethics Statement

This study was conducted with approval from both the Medical Research Ethics Committees of the Ministry of Health, Malaysia and the Faculty of Medicine and Health Sciences, Universiti Putra Malaysia, Malaysia. Written informed consent was obtained from each of the study participants.

### Study population

Sample size was calculated using the formula adopted by Schlesselman [35] on matched case-control study using a power (1-β) of 90 percent with α set at 0.05. Estimated proportion of exposed cases and controls in target population were adapted from Huang et al [36]. To detect a minimum effect size of odds ratio of 2, at least 310 matched pairs were needed. Inclusion criteria for cases include those who were histologically confirmed NPC patients diagnosed from year 2007 onwards in two public hospitals. Cases below 18 years of age or who were seropositive for Hepatitis B and C were excluded. Individuals without prior history of cancers were recruited as controls from the same hospitals and were age, sex and ethnicity matched to the cases. Only individuals recruited from the same public hospitals as the cases are eligible controls. 356 case-control pairs were available for analysis using conditional logistic regression.

### Isolation of genomic DNA and genotyping

**Fig 1:**
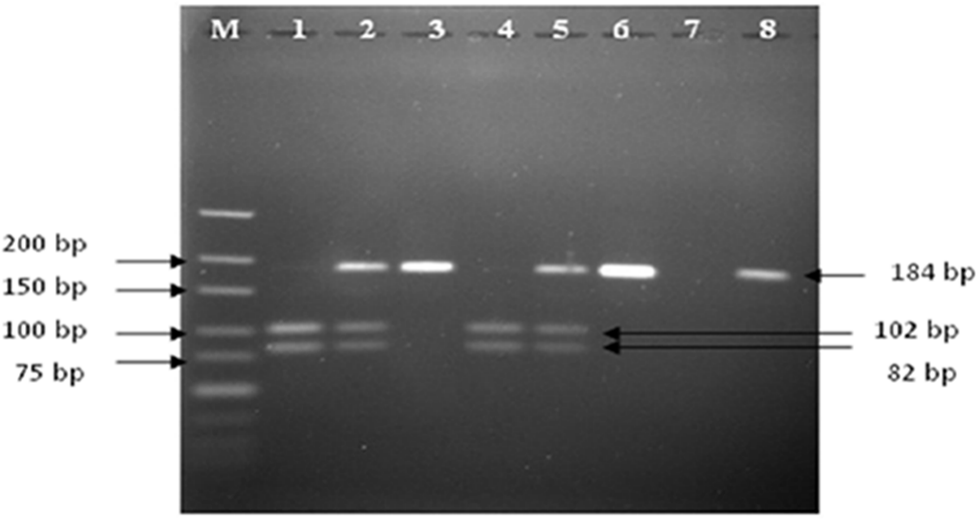
Gel electrophoresis of PCR-RFLP products for representative blood samples for the XPD Lys751Gln polymorphism. Lane M represents low range DNA ladder marker (Fermentas), lanes 1 and 4 represent Lys/Lys genotype (102 bp and 82 bp), lanes 2 and 5 represent Lys/Gln genotype (184bp, 102 bp and 82 bp), lanes 3 and 6 represent Gln/Gln genotype (184 bp), lane 7 represents negative control (RFLP reaction without PCR product) and lane 8 represents negative control (RFLP reaction without restriction enzyme, *MboII*).

Each subject donated 2.0 mL of whole blood stored in EDTA tubes at -70°C. Genomic DNA was later extracted from the blood samples using QIAamp DNA Mini and Blood Mini kit (QIAgen, USA). Polymorphism of XPD K751Q (rs13181) was detected using polymerase chain reaction—restriction fragment length polymorphism (PCR-RFLP) assay. The sequences of primers used to amplify the polymorphism of XPD K751Q were adopted from Duell et al [37]. Forward and reverse primers used were 5′-CCC CCT CTC CCT TTC CTC TG-3′ and 5′-AAC CAG GGC CAG GCA AGA C-3′ respectively. PCR-RFLP assay for the selected polymorphism is shown in Table 1. Digested PCR products were resolved on 3.0% (w/v) agarose gel. As shown in Fig 1, samples were identified as homozygous Lys/Lys (wildtype) if two PCR bands were observed at 102 bp and 82 bp. For heterozygous Lys/Gln genotype, three PCR bands were observed at 184 bp, 102 bp and 82 bp. Homozygous Gln/Gln (variant) genotype was recorded if a single PCR band was observed at 184 bp. 10% of the total samples were randomly chosen for DNA sequencing analysis to verify the PCR-RFLP results.

**Table 1:** PCR-RFLP assay of XPD K751Q polymorphism.

PCR reaction mixture	Final volume: 25 μl consisted of ~ 100ng of DNA isolated from whole blood, 5 μl of 5X PCR buffer, 2.0μl of MgCL_2_ (25mM), 0.5 μl of the mixture of dNTP (10mM), 0.5 μl of forward and reverse primers (10 μM), 0.625 U (5 U/μl) of GoTaq DNA polymerase and nuclease free-water (Promega, USA).
PCR condition	Initial denaturation was at 94°C for 5 minutes followed by 35 cycles of denaturation at 94°C for 40 s, annealing at 56.2°C for 30s, extension at 72°C for 30s and final extension at 72°C for 5 minutes.
RFLP reaction mixture	Final volume: 20 μl consisted of ~ 200 ng of purified PCR product, 2 μl of 10X NEBuffer 4, 2 μl of MbOII (5U/μl) and nuclease-free water.
RFLP condition	Incubate at 37°C for 2 hours followed by 15 minutes at 65°C.

### Statistical analysis

Relative frequencies were used to describe variables studied including socio-demographic and exposure data using SPSS version 21. Deviation from Hardy-Weinberg equilibrium (HWE) was tested using Court Lab Calculator on controls [38]. The association between potential confounders (gender, ethnicity, cigarette smoking, salted fish and alcohol consumption) and genotype was explored for cases and controls using χ^2^ analysis. Conditional logistic regression using STATA 10 was used to estimate adjusted odds ratios and their 95% confidence intervals to determine association between XPD K751Q polymorphism and risk of NPC, controlling for cigarette smoking, salted fish and alcohol consumption.

## Results

Distributions of age, gender and ethnicity were equal in both cases and controls. The mean age for both cases and controls was 53.17 years while the male to female ratio was 3.44: 1. 70.2% were ethnic Chinese while 28.4% were Malays. The distribution of cigarette smoking, salted fish and alcohol consumption among study subjects are shown in Table 2. Genotype frequencies of XPD K751Q polymorphism in cases were 305 (85.7%) homozygous Lys/Lys, 49 (13.8%) heterozygous Lys/Gln and 2 (0.5%) homozygous Gln/Gln. As for the controls, genotype frequencies were 283 (79.5%), 64 (18.0%), and 9 (2.5%) respectively. Lys/Lys genotype was more prevalent in both cases (85.7%) and controls (79.5%) compared to Gln/Gln genotype. This finding is consistent with the NCBI SNP database (rs13181) indicating that Lys/Lys is the wildtype [39]. None of the associations between factors (cigarette smoking, salted fish and alcohol consumption, gender and ethnicity) and XPD genotypes are significant. NPC cases were more likely to ever consume salted fish compared to controls (OR = 1.75, 95% CI = 1.23–2.51, p = 0.002). Individuals with previous history of smoking were also at higher risk of NPC (OR = 1.74, 95% CI = 1.20–2.52, p = 0.003). No significant difference was found between NPC cases and controls for alcohol consumption.

**Table 2:** Characteristics of the nasopharyngeal carcinoma cases and controls.

Characteristics	Cases (%) N = 356	Controls (%) N = 356
Age (mean±SD), years	53.17±11.42	53.17±11.44
Gender		
Male	276 (77.5%)	276 (77.5%)
Female	80 (22.5%)	80 (22.5%)
Ethnicity		
Chinese	250 (70.2%)	250 (70.2%)
Malay	101 (28.4%)	101 (28.4%)
Others	5 (1.4%)	5 (1.4%)
Salted fish consumption		
Never	84 (23.6%)	123 (34.6%)
Ever	272 (76.4%)	233 (65.4%)
Cigarette smoking		
Never	172 (48.3%)	212 (59.6%)
Ever	184 (51.7%)	144 (40.4%)
Alcohol consumption		
Never	191 (53.7%)	217 (61.0%)
Ever	165 (46.3%)	139 (39.0%)

**Fig 2:**
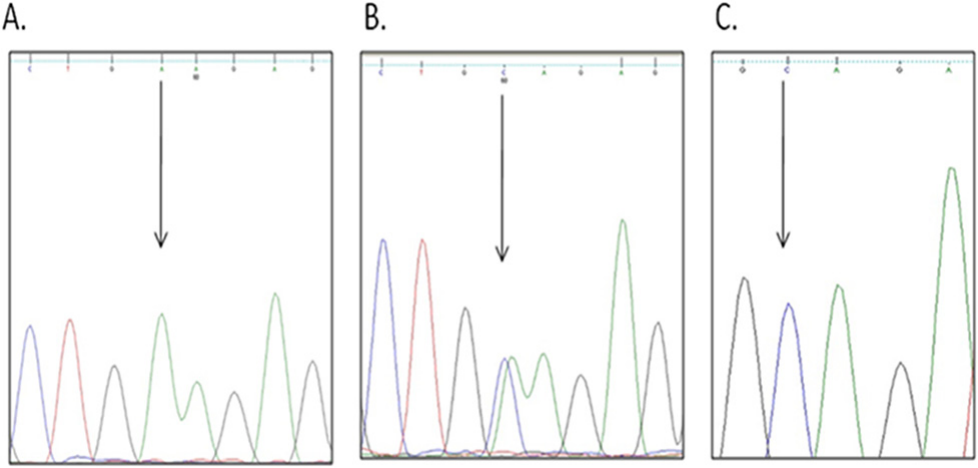
Partial sequence chromatograms of Lys751Gln polymorphism from study subjects. Arrow indicates the location of the nucleotide changes. Partial sequence chromatogram (A) represents Lys/Lys genotype, partial sequence chromatogram (B) represents Lys/Gln genotype and partial sequence chromatogram (C) represents Gln/Gln genotype.

Genotype and allelic frequencies of XPD K751Q polymorphism of controls were in Hardy-Weinberg equilibrium (p>0.05) (Table 3). There was 100% concordance of the 10% of samples sent for DNA sequencing with results obtained from PCR-RFLP assay. Partial chromatograms showing exact XPD K751Q polymorphism are presented in Fig 2. To determine the effect of K751Q polymorphism on NPC risk, a recessive model (combination of heterozygous Lys/Gln and homozygous Gln/Gln) was used due to small numbers in the Gln/Gln genotype. Homozygous Lys/Lys was compared against the combined recessive model as reference group and was found to significantly increase odds of NPC (OR = 1.58, 95% CI = 1.05–2.38, p = 0.028) after adjusting for cigarette smoking, salted fish and alcohol consumption (Table 4).

**Table 3:** Allelic and genotype frequencies of XPD K751Q polymorphism (Hardy-Weinberg equilibrium test).

	Controls (%) N = 426	Χ^2^ value	P value
Genotype			
Lys/Lys (AA)	330 (77.4%)	3.57	0.06
Lys/Gln (AC)	85 (20.0%)		
Gln/Gln (CC)	11 (2.6%)		
Allele			
Lys (A)	745 (87.4%)		
Gln (C)	107 (12.6%)		

**Table 4:** Frequency of XPD K751Q recessive model genotypes and association with NPC.

	Cases (%) N = 356	Controls (%) N = 356	Adjusted^c^ OR^a^ (95% CI^b^)	P value
Recessive model				
Lys/Lys	305 (85.7%)	283 (79.5%)	1.58 (1.05–2.38)	0.028
Lys/Gln + Gln/Gln	51 (14.3%)	73 (20.5%)	1	-
Salted fish consumption				
Never	84 (23.6%)	123 (34.6%)	1	-
Ever	272 (76.4%)	233 (65.4%)	1.76 (1.23–2.51)	0.002
Cigarette smoking				
Never	172 (48.3%)	212 (59.6%)	1	-
Ever	184 (51.7%)	144 (40.4%)	1.74 (1.20–2.53)	0.003
Alcohol consumption				
Never	191 (53.7%)	217 (61.0%)	1	-
Ever	165 (46.3%)	139 (39.0%)	1.31 (0.89–1.91)	0.170

^a^OR: odds ratio

^b^CI: confidence interval

^c^Adjusted: age, gender, ethnicity, salted fish consumption, cigarette smoking and alcohol consumption

## Discussion

In Malaysia, NPC was the 4^th^ most frequent cancer in 2011 [3]. In general, Cantonese-speaking individuals from Southern China have higher incidence rate of NPC compared to other ethnic groups [40]. Although offspring of Chinese origin who have migrated to western countries were observed to have progressively lower risk of NPC, incidence of NPC remained higher in these migrants compared to indigenous populations [40]. Similarly, results from the present study showed that majority of cases were of Chinese origin (70.2%). NPC cases in this study showed male to female ratio of 3.44:1 consistent with previous reports of males having 2–3 fold higher incidence of NPC compared to females [2]. Genetic susceptibility alone may be inadequate to explain the incidence of NPC. Inclusion of environmental factors in the causal model is crucial to better understand NPC carcinogenesis [41].

Salted fish consumption was implicated in increasing NPC risk in past studies [42–44]. In our study, subjects who consumed salted fish were found to be more susceptible to NPC (OR = 1.75, 95% CI = 1.23–2.51, p = 0.002). The causal role of cigarette smoking in carcinogenesis of various cancers has long been implicated in previous studies. Individuals who smoked were more susceptible to lung, bladder and nasopharyngeal carcinoma [45–47]. Subjects with smoking history in our study have a higher risk of NPC (OR = 1.74, 95% CI = 1.20–2.52, p = 0.003). Both these environmental factors mentioned are capable of inducing DNA damage [48–49]. Cigarette smoking-related carcinogens including polycyclic aromatic hydrocarbons (PAH) and N-nitrosamines have been shown to cause bulky DNA adducts [50]. Salted fish bought from regions with highest NPC mortality also contained high levels of N-nitrosamines [51]. NER is the main DNA repair mechanism responsible for removing bulky DNA adducts induced by the aforementioned environmental carcinogens [48]; optimal NER activity protects living cells from DNA damage. Lower than average NER activity due to interference by DNA polymorphisms in NER-related genes with constant challenges from environmental carcinogens could lead to higher NPC incidence [52].

After adjusting for effects of environmental factors, XPD homozygous wildtype Lys/Lys genotype was associated with higher odds of NPC (OR = 1.58, 95% CI = 1.05–2.38, p = 0.028). This observation contradicts some of the conclusions drawn by previous studies, in which homozygous Gln/Gln was the genotype implicating risk of various cancers. Benascu et al. [53] reported that homozygous Gln/Gln was associated with higher risk of chronic myeloid leukemia (OR = 2.37; 95% CI = 1.20–4.67, p value = 0.016). Huang et al. [36] showed that Gln allele was associated with a significantly higher risk of esophageal squamous cell carcinoma while Gln/Gln genotype carriers are associated with increased risk of digestive tract cancer. However, evidence from other studies showed that the Lys allele increased risk in various cancers. Yang et al. [54] found Lys allele to be associated with increased risk of NPC in Sichuan population. In another study in West Bengal by Banerjee et al. [55], Lys/Lys genotype was shown to be associated with increased risk (OR = 4.77, 95% CI = 2.75–8.23) of developing arsenic-induced premalignant hyperkeratosis, which is a precursor lesion of arsenic-induced skin cancer. Lys allele was also implicated in increasing risk of oral leukoplakia and cancer (OR = 1.6, 95% CI = 1.1–2.3) [56]. Ozcan et al. [57] found the Lys/Lys genotype increased risk of early relapse in hematological malignancies (OR = 13.12, 95% CI = 1.09–157.76). Lunn et al. [58] compared 61 Caucasian women with and without a family history of breast cancer and showed that Lys/Lys genotype was associated with impaired repair of X-ray-induced DNA damage as evidenced by a higher level of chromatid aberrations compared to individuals with Gln alleles.

Several postulations have been put forward in order to explain the effect of XPD K751Q polymorphism on NPC risk. Firstly, Friedberg E. [59] proposed that an A to C nucleotide change at XPD codon 751 causes an amino acid substitution from Lysine to Glutamine that results in alteration of the electronic configuration of amino acid from acidic to basic. Kuper et al. [13] showed that XPD helicase activity is deficient without proper XPD-p44 interaction leading to impaired unwinding of DNA during NER process. Secondly, Sturgis et al. [60] reported that XPD protein expression might be altered due to the close proximity of K751Q polymorphism’s location to the poly (A) signal made up of AAUAAA hexamer, which is recognized by components of cleavage and polyadenylation complex [61] and is the key trigger for pre-mRNA 3’ end processing. Polyadenylation is an important process in living cells because poly (A) tail protects RNA from enzymatic degradation. A nucleotide change in K751Q could lead to failure in recognizing poly (A) signal by polyadenylation complex resulting in a lower than normal DNA repair capacity from diminished XPD gene expression due to less XPD mRNA that will escape the highly active enzymatic degradation [62].

The molecular basis of the association between XPD K751Q polymorphism and NPC needs to be further elucidated. Differences in the integrity and functionality of TFIIH complex between Lys/Lys and Gln/Gln genotype and involvement of other genes in NER mechanism (p44 protein) could provide evidence needed to explain the association. Other DNA repair pathways (base excision repair, BER) and other cell signaling pathways such as Wnt, MAPK and PI3K-Akt pathway are also implicated in NPC carcinogenesis [63]. Future research investigating crosstalk between DNA repair and other pathways are warranted to better understand NPC carcinogenesis.

## Supporting Information

S1 Dataset Minimal dataset used in analyses. (XLSX)
